# Analysis of simultaneous MEG and intracranial LFP recordings during Deep Brain Stimulation: a protocol and experimental validation

**DOI:** 10.1016/j.jneumeth.2015.11.029

**Published:** 2016-03-01

**Authors:** Ashwini Oswal, Ashwani Jha, Spencer Neal, Alphonso Reid, David Bradbury, Peter Aston, Patricia Limousin, Tom Foltynie, Ludvic Zrinzo, Peter Brown, Vladimir Litvak

**Affiliations:** a Wellcome Trust Centre for Neuroimaging, 12 Queen Square, London, UK; b Nuffield Department of Clinical Neurosciences, John Radcliffe Hospital, Oxford, UK; c Sobell Department of Motor Neuroscience and Movement Disorders, Institute of Neurology, Queen Square, London, UK

**Keywords:** Magnetoencephalography (MEG), Local Field Potential (LFP), Deep Brain Stimulation (DBS), Parkinson's disease

## Abstract

**Background:**

Deep Brain Stimulation (DBS) is an effective treatment for several neurological and psychiatric disorders. In order to gain insights into the therapeutic mechanisms of DBS and to advance future therapies a better understanding of the effects of DBS on large-scale brain networks is required.

**New method:**

In this paper, we describe an experimental protocol and analysis pipeline for simultaneously performing DBS and intracranial local field potential (LFP) recordings at a target brain region during concurrent magnetoencephalography (MEG) measurement. Firstly we describe a phantom setup that allowed us to precisely characterise the MEG artefacts that occurred during DBS at clinical settings.

**Results:**

Using the phantom recordings we demonstrate that with MEG beamforming it is possible to recover oscillatory activity synchronised to a reference channel, despite the presence of high amplitude artefacts evoked by DBS. Finally, we highlight the applicability of these methods by illustrating in a single patient with Parkinson's disease (PD), that changes in cortical-subthalamic nucleus coupling can be induced by DBS.

**Comparison with existing approaches:**

To our knowledge this paper provides the first technical description of a recording and analysis pipeline for combining simultaneous cortical recordings using MEG, with intracranial LFP recordings of a target brain nucleus during DBS.

## Highlights

• Setup for MEG and intracranial recordings during Deep Brain Stimulation is described.
• Phantom experiment showed correct recovery of oscillatory sources despite artefacts.
• The method is applied to real data from a patient with Parkinson's Disease.
• Cortico-subthalamic coherence profiles on and off stimulation were comparable.

## 1 Introduction

Deep Brain Stimulation (DBS) is a way of treating neurological and psychiatric disorders that involves electrical stimulation of subcortical brain regions through chronically implanted macro-electrodes. One condition in which DBS therapy has proven to be particularly effective is Parkinson's Disease (PD) (Deuschl et al., 2006). In PD, the most commonly targeted brain region for DBS is the subthalamic nucleus (STN), but other less frequently implanted sites include the thalamus, the pedunculopontine nucleus and the globus pallidus (Lukins et al., 2014).

Each DBS procedure presents a unique opportunity to gain valuable translational insights about electrophysiological brain activity in pathological disease states (Oswal et al., 2013a). DBS electrodes are sometimes externalised post-operatively to verify correct electrode placement prior to stimulator implantation. This enables local field potential recordings from the target nuclei. As a result, it has been possible to gain valuable information about the clinical and functional correlates of oscillatory activity within the STN in PD. An additional role of such recordings has been to shed light on the local therapeutic mechanisms of DBS. For example, it has been possible to show that DBS reduces beta band activity within the STN (Eusebio et al., 2011, Rossi et al., 2008), and furthermore that the extent of beta reduction correlates with clinical improvement (Kühn et al., 2009, Kühn et al., 2006, Ray et al., 2008, Chen et al., 2010). Such insights are being translated into improved clinical therapies, as highlighted by a seminal study of adaptive DBS in the Parkinsonian non-human primate (Rosin et al., 2011), and recent pilot work also suggesting that adaptive DBS, triggered to beta band amplitude, may potentially be better than conventional DBS at ameliorating Parkinsonian symptoms in patients (Little et al., 2013). Despite these promising early insights, many questions remain yet to be answered about the neuromodulatory effects of DBS on long-range brain networks. Magnetoencephalography (MEG) may provide a powerful approach for imaging brain networks during DBS.

Previous work using simultaneous MEG and resting intracranial LFP recordings has shown the existence of two spatially and spectrally distinct networks between the STN and cortical regions in PD (Litvak et al., 2010, Litvak et al., 2011a, Hirschmann et al., 2011). An alpha band network exists between the STN and temporo-parietal and brainstem regions, whilst a beta band network exists between the STN and motor/premotor regions. Furthermore, the initiation of movement is accompanied by a dopamine dependent reduction in coherence in the alpha network and a concomitant emergence of gamma band synchrony between the STN and primary motor cortex (Oswal et al., 2013b, Litvak et al., 2012). The identified resting state networks may play an important role in the pathophysiology of PD, highlighted by the fact that dopamine dependent modulations in their activities correlate with dopamine related improvements in clinical scores. Although such correlations by no means imply causation, their presence is highly informative. It is worthwhile noting that even in the absence of DBS, these recordings are severely contaminated by the presence of high amplitude artefacts related to the presence of ferromagnetic extension wires from the DBS electrodes to the recording equipment. However, source space analysis using beamformers enables effective suppression of such artefacts, allowing for valid physiological inferences to be made (Litvak et al., 2010, Oswal et al., 2014). Non-ferromagnetic extension wires have recently been made available, but they are not approved for clinical use in the UK at present (Hirschmann et al., 2011).

Although it has been possible to simultaneously stimulate and record from the STN, and also to record from the STN during concurrent MEG, no methods have as yet been developed to combine these two approaches and allow simultaneous MEG and intracranial LFP recordings during DBS. The utility for such recordings is clear, since they would enable the characterisation of both local effects of DBS and of the effects of DBS on connectivity between target nuclei and distal brain regions. Such information could benefit current understanding of the therapeutic mechanisms of DBS in addition to informing future developments in DBS technologies.

In this paper we describe the experimental setup for simultaneous MEG and intracranial LFP recordings during DBS. We also detail the analysis procedure making it possible to recover coherence between the LFP and the MEG in the presence of stimulation, despite artefacts due to ferromagnetic extension wires, and those due to DBS currents that will not be obviated by the use of non-ferromagnetic extension wires. The proposed methods are validated using a dipole phantom and applied to data from a single PD patient with electrodes in the STN.

## 2 Methods

### 2.1 Simultaneous MEG and LFP recordings

All the MEG recordings described in the present paper were performed using a CTF 275-channel MEG system (CTF/VSM MedTech, Vancouver, Canada). An important advantage of this system is the high dynamic range of its sensors and their robustness to strong external interferences.

In our previous studies we used the EEG system integrated in CTF-MEG to record the intracranial data (Litvak et al., 2011a, Litvak et al., 2012, Oswal et al., 2013b). Our rationale for moving away from this approach was that the recording equipment was not isolated from the mains power supply and thus did not comply with newer and more rigorous local safety standards. The approach we use in the present study is to record LFP and other electrophysiological measurements from the patient using a battery-powered and optically isolated BrainAmp system (Brain Products GmbH, Gilching, Germany). The challenge that this approach poses is fusing the EEG and MEG data with minimal timing distortions. For this purpose we propose to use a synchronisation signal recorded on both systems. The optimal type of signal is random white noise because it can only be matched in a unique way.

Any noise generator capable of producing wide-band noise with an appropriate amplitude can be used. Our generator was built utilising an 8.2 V Zener diode biased close to its avalanche region. This was achieved using a variable potentiometer, while an oscilloscope was used to ensure the maximum noise was produced. The noise was amplified to 500 mV peak-to-peak range. To control the exact output amplitude a variable resistor on the output was included. Note that connecting the noise generator with cables to both the MEG and the EEG amplifier would create a breach in the optical isolation and defeat its purpose. Fortunately, the BrainAmp system provides a solution by combining two optically isolated amplifiers into one system with synchronous sampling—accordingly one of these amplifiers was used to record the noise signal, whilst the other was used to record physiological activity.

**Fig. 1:**
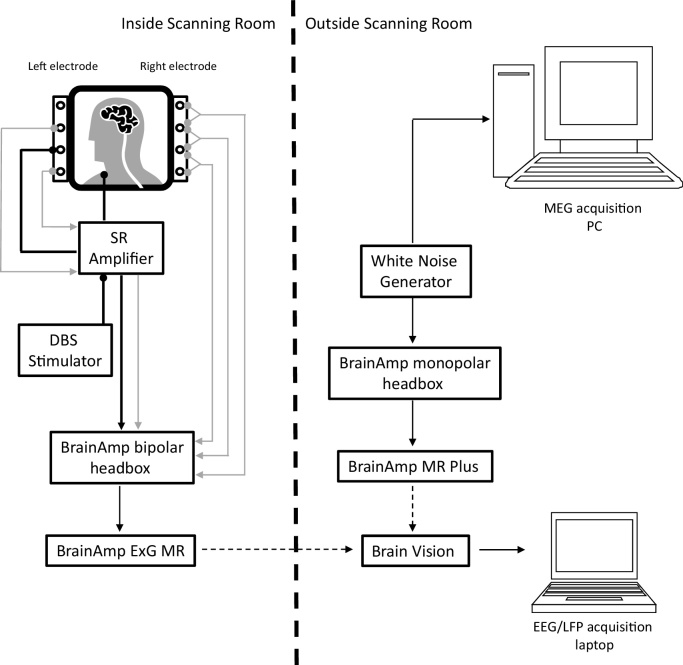
Schematic showing our set up for a patient recording within MEG. A bipolar LFP between contacts 0 and 2 of the DBS electrode (see grey lines originating from left electrode) is recorded during concurrent DBS by the stimulation record (SR) amplifier. DBS is given in a monopolar fashion between contact 1 of the DBS electrode and a reference attached to the patient's clavicle (see black lines ending with circles). The SR amplifier outputs the recorded LFP and the stimulation signal (grey arrowed line and black arrowed line) to a bipolar BrainAmp headbox. The latter is also used to record bipolar LFPs from the side not being stimulated (see grey arrowed lines originating from the right electrode). Black dashed arrows depict optic fibre cables, which serve to optically isolate the patient from a mains power source (see methods section). The timings of signals recorded from the MEG and the LFP acquisition laptop are synchronised through the independent recording of a single white noise source on the two systems (see methods for further details).

Fig. 1 details our experimental set up. We note that it is possible to record the white noise and the physiological signals (LFP, EEG, EOG, EMG) in either a monopolar or a bipolar fashion. For the purposes of our experiment we recorded physiological signals bipolarly (using an 8 channel bipolar ExG BrainAmp amplifier), since our stimulation-record amplifier was designed to give bipolar outputs. The white noise was recorded using a 32 channel monopolar BrainAmp MR plus amplifier. Our set up was approved by the MEG safety board of the Wellcome Trust Centre for Neuroimaging, following extensive in-house safety testing.

### 2.2 Simultaneous stimulation and LFP recording

In this section we detail our approach for simultaneous DBS and LFP recording during MEG. We used a purpose built stimulation-record amplifier that was a variant of the design used in previous studies not involving MEG (Eusebio et al., 2011, Little et al., 2013, Rossi et al., 2007).

This design is based on the idea that when stimulating one of the two middle contacts (contact 1 or 2) monopolarly while recording from the two adjacent contacts of the DBS electrode (0 and 2 or 1 and 3, respectively) one can use the common mode rejection property of the front stage differential amplifier to reduce the DBS artefact and line noise. Both these signals are seen similarly by the recording contacts. The stimulation-record amplifier is used in combination with a clinically approved external DBS stimulator (type 3628, Medtronic Inc., Minneapolis, MN) connected to the intracranial electrode via the amplifier circuit.

The amplifier consists of the following components:

(1) Front stage amplifier with a gain of ×10 (any higher could saturate the amplifier due to DC voltages on the contacts).
(2) DC blocking component, to remove any offsets that could cause saturation of the subsequent stages.
(3) Low pass filter at 40 Hz to further reduce the line noise and DBS artefacts (when 130 Hz DBS is delivered). This filter is based on a Sallen–Key low pass design with −12 db per stage. Two stages were used giving −24 db of attenuation for each doubling of frequency after 40 Hz, which is in most cases sufficient to recover the frequencies of interest.
(4) In addition to the LFP from the stimulated location the amplifier also generates a copy of the stimulation train which can later be used e.g., for segmenting the data. This is achieved by connecting the stimulator signal to an opto-isolator in parallel with the patient. The stimulator pulse of about 60 μs is too fast for sampling by the EEG amplifier requiring the use of a 555 timer circuit to stretch the pulse to approximately 2 ms. The pulse is then reduced in amplitude to a few mV.

The amplifier produces two bipolar output signals compatible with the BrainAmp ExG box: LFP and stimulation train copy. Consequently during blocks of recordings with DBS on, we were able to record both a single bipolar LFP from the side being stimulated, and 3 bipolar LFPs from the contralateral side. Further details regarding this amplifier are available from the authors on request.

### 2.3 Experimental protocol—phantom

**Fig. 2:**
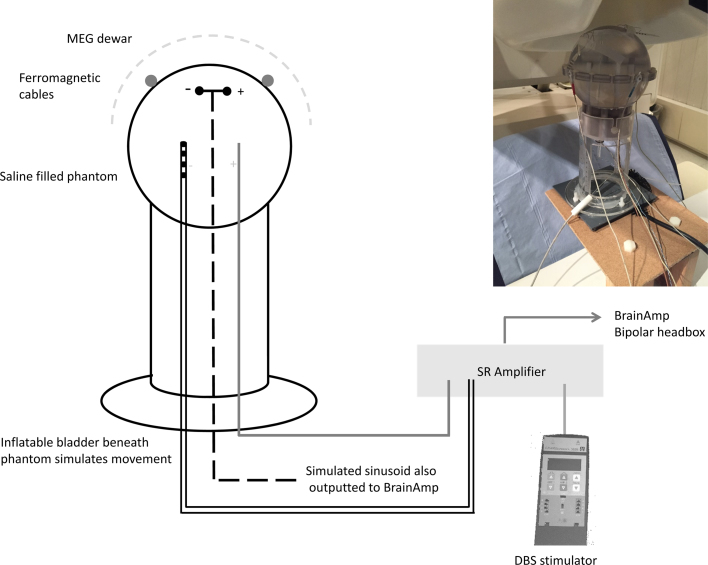
Schematic showing the phantom recording setup within MEG. The phantom was filled with saline and positioned in the MEG Dewar. Three electrodes were immersed in the phantom: (1) An electrode which simulated a dipole with a sinusoidal signal at 27 Hz (shown by the dashed black line), (2) A clinical DBS electrode (see Section 2), one contact of which was used to administer monopolar DBS at clinical settings, and (3) A second electrode for DBS such that monopolar DBS was administered between this electrode and contact 1 of the DBS electrode. In this case, contact 1 of the DBS electrode was the cathode. Note that in this schematic, the dipolar source is shown as being central within the phantom, but its exact location is shown more precisely in Fig. 7. Two ferromagnetic extension cables that are typically used in patient recordings were taped loosely to the surface of the phantom. Heartbeat artefacts (see Section 2) were simulated using an inflatable bladder that was positioned beneath the phantom and periodically inflated at a rate of 1 Hz. For the phantom recordings the stimulation-record amplifier (SR amplifier) was used in conjunction with the external stimulator to provide DBS. In patient recordings however, the SR amplifier was also used to record the STN LFP during DBS and to amplify this before outputting it to the BrainAmp device. A copy of the simulated sinusoidal signal was also outputted to BrainAmp.

To demonstrate that MEG activity coherent with a reference channel can be accurately recovered in the presence of DBS artefacts we performed an experiment with a MEG phantom. We used a CTF current dipole phantom comprising a spherical plastic container, holding saline, in which a dipolar source can be immersed (see schematic of setup in Fig. 2). The dipolar source was driven by a 27 Hz sinusoidal signal, so as to mimic activity within the physiological beta range, and its amplitude was set to 6.7 μA to create a peak in the power spectrum slightly exceeding the background noise level (see Fig. 5).

In addition to the dipolar source, additional electrodes were immersed in the phantom. This was done via openings at the bottom of the sphere that are usually used to vary the position of the dipole source. The openings were sealed around the electrodes to prevent saline leakage. One of the additional electrodes was a clinical DBS electrode, model 3389 (Medtronic Neurological Division, Minneapolis, MN) with four platinum-iridium cylindrical surfaces (1.27 mm diameter and 1.5 mm length) and a centre-to-centre separation of 2 mm. Contact 0 was the closest to the electrode tip and contact 3—the farthest. To emulate monopolar stimulation we immersed an additional anodal reference electrode in the phantom. Accordingly, in our recordings monopolar DBS was administered between contact 1 of the DBS electrode and the anode. Bipolar DBS in contrast is administered between adjacent contacts of the DBS electrode. In our phantom recordings, bipolar stimulation was administered between contacts 0 (cathode) and 1 (anode) of the DBS electrode. Note that in our recordings with the phantom, we tested monopolar and bipolar DBS separately in order to characterise the different kinds of sensor artefacts produced by each of these stimulation regimes. All analyses of coherence, however, are presented only during monopolar DBS.

A copy of the sinusoidal input to the current dipole was recorded bipolarly with the BrainAmp amplifier system as would be done with the intracranial LFP. Note that although in the monopolar arrangement we could also record the potential difference between contacts 0 and 2 of the DBS electrode, using it as a surrogate LFP would not mimic the patient recording well. This is because the dipole signal would only be picked up by volume conduction in water from a distant source, whereas in the real patient the LFP signal is picked up from neurons surrounding the electrode. Therefore, we did not record from the stimulating electrode in the phantom experiment.

Three Head Position Indicator (HPI) coils were attached to the phantom in order to facilitate co-registration between the MEG co-ordinate system and the phantom. The coils were driven with sinusoidal signals at the frequencies of 1425 1475 and 1525 Hz. The phantom was raised into the MEG helmet and whilst the dipolar source was active, three minute recordings were performed for each of the following experimental conditions: (1) No DBS, (2) Monopolar DBS at 130 Hz (3) Monopolar DBS at 20 Hz (4) Bipolar DBS at 130 Hz (5) Bipolar DBS at 20 Hz.

DBS was administered with a Medtronic external stimulator (type 3628) via the stimulation-record amplifier with a pulse width of 60 μs and amplitude of 3 V. Our measurements showed that the current flowing through the phantom for these parameters was 6 mA which corresponds to charge of 0.36 μC/phase and charge density of 6 μC/cm^2^ x phase. These values are similar to those measured for real DBS in patients (Kuncel and Grill, 2004). The rise time of the pulse was ∼1 μS.

Although stimulation at 130 Hz produces the greatest clinical benefit, we also explored the effect of stimulating at 20 Hz as a means of probing beta band resonance within the cortico-subthalamic nucleus network.

In order to make our phantom simulation as realistic as possible, we added two additional and related artefacts which are seen in recordings with patients. Firstly we simulated low amplitude movements to replicate arterial pulsations and slight head movements resulting from each heartbeat. These movements were created by placing an inflatable balloon under the phantom, which was periodically inflated with air at a rate of 60 times per minute. Air inflation was performed using custom made electronics, connected to a pressurised air supply which was controlled using a MATLAB script. We ensured that the resulting movements of the phantom were similar in terms of their magnitude to head movements that are observed in patient recordings by ensuring that the channel spectra were comparable (see Results section). To simulate the second type of artefact, we placed two ferromagnetic extension wires which were identical to those used in patient recordings on the spherical surface of the phantom. We have previously shown that movements of the ferromagnetic wires are related to arterial pulsations and that the interaction of these two phenomena is major source of artefacts in recordings without stimulation (Litvak et al., 2010).

### 2.4 Patient experiment

We present data from a 43 year old male patient with a nine year history of PD, who underwent implantation of bilateral DBS electrodes in the STN. The recordings were approved by the local ethics committee, and the patient gave written informed consent prior to participation. The permanent DBS macroelectrode implanted in the STN was model 3389 as in the phantom experiment (see above). Further details of the operative procedure can be found in (Foltynie et al., 2011, Foltynie and Hariz, 2010). The locations of the electrodes were confirmed following implantation with immediate postoperative fast spin-echo T2-weighted magnetic resonance imaging (MRI) with a Leksell frame still in situ. The patient's preoperative T2-weighted MRI was used to construct a head model (see source space analysis for further details). Electrode extension cables were externalized through the scalp to enable recordings prior to connection to a subcutaneous DBS pacemaker, implanted in a second operative procedure 7 days later.

### 2.5 Experimental protocol–patient recordings

Recordings were performed after overnight withdrawal from dopaminergic medication. The patient was requested to keep his eyes open and to stay still during recordings. A neurologist was present inside the magnetically shielded scanner room at all times during the recordings, to monitor the wellbeing of the patient and to administer DBS.

The experiment started with a 4 min resting block during which data were collected directly via BrainAmp without the stim-record amplifier. The stim-record amplifier and external stimulator were then added to the setup and two additional recording runs were performed. Two stimulation conditions were tested independently for the right and left STNs: 0 Hz (no DBS), and 130 Hz monopolar DBS. The right and left STNs were stimulated separately in different recording runs. Each recording run was 7 min long and included two conditions, each lasting 3 min, separated by a 1 min interval for washout of the effect of the first condition. Stimulation conditions were assigned to runs in random order. We also tested DBS at 5 Hz and 20 Hz in the same experiment but the data are not presented here.

Monopolar DBS, between contact 1 and an external reference applied to the patient's clavicle was administered by the neurologist. The stimulation pulse width and amplitude were 60 μs and 3 V respectively. At the onset of DBS, the stimulation voltage was increased slowly, in increments of 0.5 V, whilst checking for clinical improvement and for the presence of any stimulation related side effects. Prior to experimentation, clinical assessment of the patient confirmed an improvement in motor (Part III) Unified Parkinson's Disease Rating Scale (UPDRS) scores with monopolar DBS at 3 V and 130 Hz.

### 2.6 Electrophysiological recordings

MEG data were sampled at 2400 Hz and stored to disk for subsequent offline analyses. Head location was continuously monitored throughout the experiment. For runs with two active DBS conditions a recording of ∼1 min without DBS was added at the beginning to aid extraction of head location.

LFP recordings from both STNs were simultaneously collected using the BrainAmp system. Patient data were collected via the 8-channel bipolar headbox and BrainAmp ExG amplifier, as shown in Fig. 1. Eight intracranial LFP channels representing contacts 0-3 of each DBS macroelectrode were converted using bridge connectors into a bipolar montage, between adjacent contacts. This resulted in 3 bipolar channels (0–1, 1–2, 2–3) recorded from each side and an additional channel recording the potential difference between contacts number 0 on the two sides (L0-R0) to enable if necessary converting the data to monopolar recordings referenced to the contralateral side.

After the initial resting recording the right side channels and L0-R0 channel were disconnected and bipolar channels #1 and #2 of the headbox were used for the stimulus train copy and the LFP recording from the stimulation-record amplifier. Channels #3 and #4 were left unused and channels #5–7 recorded from the unstimulated side. Initially this was the left side, but after the first two stimulation runs the connectors were swapped so that left became the stimulated side and right the unstimulated side.

LFP recordings were high pass filtered at 1 Hz in the hardware so as to avoid amplifier saturation due to large DC offsets. The data were sampled at 2500 Hz which was the closest available sampling rate to the one used in MEG. LFPs were recorded onto a laptop that was optically isolated from the BrainAmp hardware. Random noise from the noise generator was recorded simultaneously on the BrainAmp system via the 32-channel monopolar BrainAmp MR Plus amplifier and the MEG via one of the Analogue to Digital Converter (ADC) channels (see Fig. 1).

### 2.7 Offline data analysis

All analyses were performed using custom MATLAB scripts in combination with the SPM12 (http://www.fil.ion.ucl.ac.uk/spm/software/, Litvak et al., 2011b), Data Analysis in Source Space (DAiSS, http://www.fil.ion.ucl.ac.uk/spm/ext/#DAiSS) and Fieldtrip (http://www.ru.nl/neuroimaging/fieldtrip/, Oostenveld et al., 2011) toolboxes.

### 2.8 Determining head location

In our previous paper (Litvak et al., 2012) we described a way of dealing with intermittent problems with head tracking based on the fact that the measured distances between the HPI coils should stay constant (up to the measurement precision) when the head location is correctly measured. Here we describe a further development of this idea aimed at maximal usage of head tracking information recorded in the same session. The CTF system records the head location at the beginning of a run and also can be configured to record head location data continuously throughout the run. The initial measurement is controlled by the MEG operator who is shown whether the measurement is valid for all the three fiducials and if not has the possibility to wait further until a valid measurement is obtained. In our experience, obtaining valid initial measurements has been difficult due to the interferences of the ferromagnetic percutaneous wires (Litvak et al., 2010) with MEG sensors. It seems to require stable tracking for a few seconds so often a measurement that seems to be correct is still not accepted by the CTF software due to instabilities. We therefore opted not to wait too long in these cases and started the recording in order to avoid exhausting the patient.

An experiment usually produces a number of separate runs each containing an initial head location measurement (possibly invalid) and continuous head location data for the whole run, part of which might also be invalid. The idea of the analysis described below is to generate the best estimate of the head location in each run even if a large portion of the head location measurements are invalid. The analysis is based on the assumption that for valid head location measurements the pairwise distances between HPI coils will be the same (up to a small measurement error) whereas for invalid measurements they will be highly variable. Our approach had been optimised to work on a large group of recordings without concurrent DBS and was robust enough to work unmodified also with recordings including DBS despite the fact that during stimulation delivery head tracking was clearly disrupted.

We performed two passes on combined head tracking data from all runs. The aim of the first pass was to determine the distances between HPI coils. For this purpose we collected from each run two head location estimates. The first was the initial head location measurement. The second was extracted from continuous location data by computing pairwise distances between fiducials in cm, rounding to the nearest integer and finding the most common value across time (using ‘mode’ function in MATLAB). Time points for which one of the distances differed from its mode value by more than 1 cm were marked as invalid and interpolated using linear interpolation and extrapolation over the whole run. Corrected head location was then computed as the median over all points, original and interpolated.

We then examined pairwise distances between fiducials for all the collected measurements. In a typical stimulation experiment there would be 5 runs, yielding 10 measurements. Again, ‘mode’ function was used to find the most common set of distances and measurements with distances differing from the mode by more than 1 cm were marked as invalid. Averaged distances for the valid measurements were used in the second pass over the data. This pass again involved correction and interpolation of the original continuous head tracking traces with the difference being that distances for each time point were compared not to the mode of that same run but to the valid distances computed across all runs in the first pass. The rationale for that was that there might be some cases where tracking was valid only briefly so that the true values were not even most common in a run. Still if those brief moments could be identified one would get a better estimate of head location in that run than by taking it from a different run.

Finally, the original and corrected head locations were examined for the second time across all runs and their validity was determined by comparing distances to their most common values as described above. The average of all the valid locations was then computed. Each run was assigned a head location in the following order of preference. If the location computed from continuous head tracking data was valid, it was used because it best reflected the true head location during the measurement. If not, the initial measurement was used. If both run-specific measurements were not valid, the average over the valid measurements from other runs was used.

In practice, for the subject shown in the present paper, and all the subjects we studied for the group study not shown here, a run-specific head location could always be successfully recovered. Head and sensor locations were always visually inspected and compared across runs to make sure that there are no gross outliers and misregistrations. Note that our aim in this analysis was to recover a single most representative head location for each run rather than completely reconstruct the head movements during the run and compensate for them. There was no severe head movement problem in our study with the patient off dopaminergic medication. Thus, the inability to recover valid head location during active DBS is not very problematic in our case because we recorded at least 30 s of data with no stimulation in each run and recovered the head location from it.

### 2.9 Fusion of MEG and LFP data

As the first processing step the raw data files from the CTF-MEG and BrainVision systems were converted to SPM MATLAB-based format. The LFP data were downsampled to 2400 Hz, matching the sampling rate of the MEG data. The white noise recordings from the MEG and BrainVision systems were linearly de-trended prior to cross-correlating the two time series. If both recordings were valid and the MEG and LFP files were matched correctly a clear peak could be detected in the cross correlation exceeding by far the background cross-correlation values. Our criterion for detecting this peak was that the ratio of the peak value to the median of the absolute values of cross-correlation across all delays was above 25. Based on the location of the peak with respect to the zero lag we determined the offset of the two recordings. This value could be used for the initial rough alignment. By computing local cross-correlations for different parts of the recording we found that this rough alignment would result in a slow drift between the two noise traces which would typically accumulate to 0.7 samples per minute of recording. The reason for this drift could be the fact that the true sampling rates of the two recordings were slightly different. We thus refined the alignment further using the following procedure. Non overlapping 1-s segments were defined in the units of samples for the MEG data starting with the first sample. Corresponding segments were then defined for LFP data by adding the offset value determined from global cross-correlation to the MEG segment definition. MEG segments for which there was no LFP counterpart (due to asynchronous start and end of the two recordings) were discarded. Cross-correlations were then computed segment-wise between the MEG and LFP noise copies. For each segment, if a clear peak (exceeding 0.2 for normalised cross-correlation) was found in the lag range of ±20 samples (±8.3 ms) the corresponding offset was added to its LFP segment definition, so that repeating the procedure with corrected LFP segment definition would only yield peaks at zero lag.

The resulting segment definitions for MEG and LFP were then used to epoch the full MEG and LFP data. The epoched datasets were fused and then converted again into a continuous recording (which was straightforward because the segments were consecutive in time). In this new dataset MEG and LFP data were fused and aligned. We chose to perform the fusion as early as possible in the processing pipeline to be able to process our older datasets recorded with the CTF EEG system and the new fused datasets in the same way.

### 2.10 Removal of MEG jumps

Visual inspection of the individual channel data obtained during monopolar DBS revealed high amplitude jumps in some of the channels that lasted only a few samples (see Fig. 6 and Section 3). The jumps contained power at all frequencies and could not, therefore, be removed by simple filtering. Importantly, however in both stimulation conditions, the majority of channels were not affected by jumps.

Prior to further analysis, we rejected channels where the number of jumps exceeded 1000—where we defined a jump as an absolute difference in the magnetic field between adjacent samples of greater than 10^5^ fT. Channels with more than 1000 jumps in any experimental condition were removed from all experimental conditions, in order to keep the conditions comparable. This threshold for channel rejection was determined empirically as it allowed us to remove channels that were worst affected by jumps, leaving those that could be easily repaired, using an interpolation-based approach as follows. A fixed segment of data (120 ms) either side of the jump was examined and DBS stimulation pulse peaks within it were identified. The data between DBS pulse peaks greater than two pulses away from the jump on either side were averaged to produce a signal for interpolating the region contaminated with the jump. The contaminated region of data was defined as the segment of data between two DBS pulse peaks of the jump on either side. Finally, the mean of the post-jump data segment was adjusted to correspond to that of the pre-jump data, ensuring smooth fixing of the jump (see Fig. 6). Following jump correction, the data were downsampled to 300 Hz and high pass filtered above 1 Hz prior to epoching.

### 2.11 Trial definition

For phantom data each stimulation frequency was recorded in a separate run and the recording was started and stopped with DBS ongoing. Therefore, the fused data were epoched into 4 s long trials starting with the recording onset.

For patient data the situation was more complicated with stimulation parameters changing during the recording. We, therefore, used the stimulation train copy generated by the stimulation-record amplifier to segment the recording. Stimulation peaks were detected in the continuous recording and the segments where the stimulation frequency matched one of the two known frequencies for that run (or in the case of 0 Hz where there was no stimulation) were identified. Margins of 20 s were then removed from the beginning and the end of the segment to only include a stable stimulation period sufficiently distant from any parameter changes. This period was epoched into 4 s segments.

### 2.12 Sensor level analysis

**Fig. 3:**
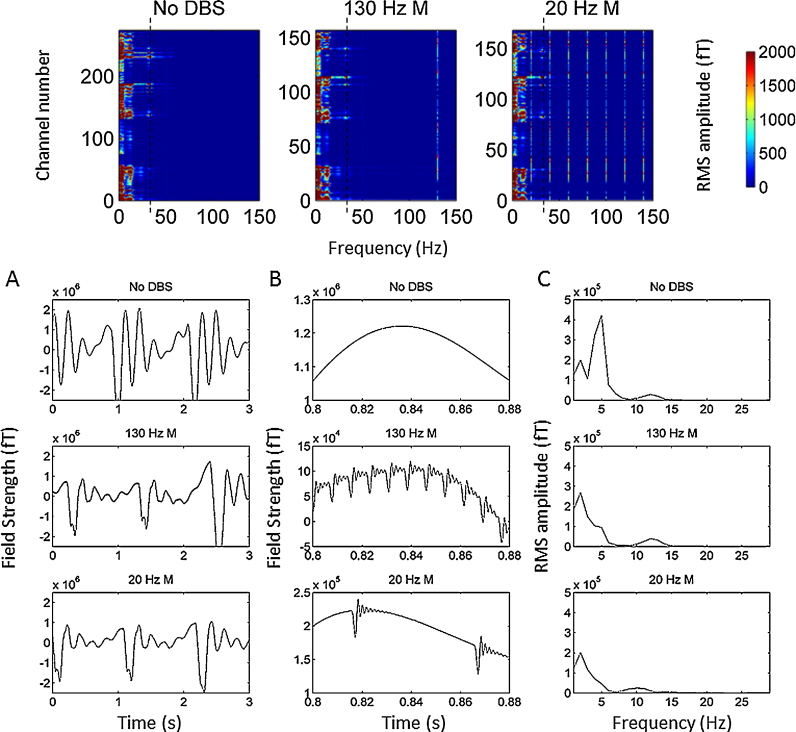
Upper panel displays individual channel spectra for the no DBS condition and the 130 Hz and 20 Hz monopolar DBS conditions. The colour bar represents RMS amplitude in femtoTesla (fT). Low frequency high amplitude artefacts (<10 Hz) affect the majority of channels. Note that the spectra for the two stimulation conditions contain fewer channels, since channels with large numbers of jumps have been rejected. The dotted black line displays a separate type of artefact at 32 Hz, which is further described in Fig. 5. In order to establish the temporal characteristics of the low frequency artefacts, single channel data are presented for a 3 s long recording period with the highest power at frequencies less than 10 Hz. Data are presented in the lower panel of Fig. 3 for the no DBS condition and the two monopolar DBS conditions. The left hand side plots (Panel A) show 3 s of data with the same y axis for comparison of the signal amplitudes. The middle plots (Panel B) show only 0.08 s of data and the y axis are varied so that the signal can be better visualised in all 3 conditions. The plots on the far right (Panel C) are the spectra of the data presented in Panel A. The effects of movements of the ferromagnetic wires which occur at a rate of approximately 1 Hz are clearly visualised in Panel A.

**Fig. 4:**
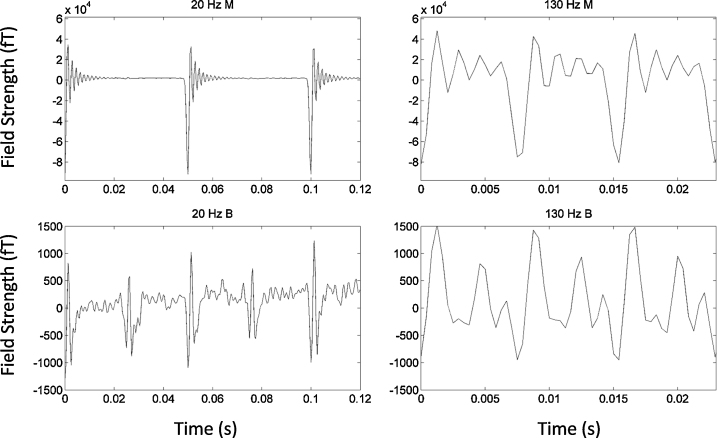
The ringing artefacts are shown for a single MEG channel following three stimulation pulses for the 20 Hz and 130 Hz monopolar and bipolar stimulation conditions. The artefacts induced by bipolar DBS are approximately 25 times lower in amplitude than those induced by monopolar DBS. Furthermore, the period between stimulation pulses is longer and therefore less influenced by ringing during 20 Hz DBS than during 130 Hz DBS (50 ms during 20 Hz and 7.7 ms during 130 Hz).

**Fig. 5:**
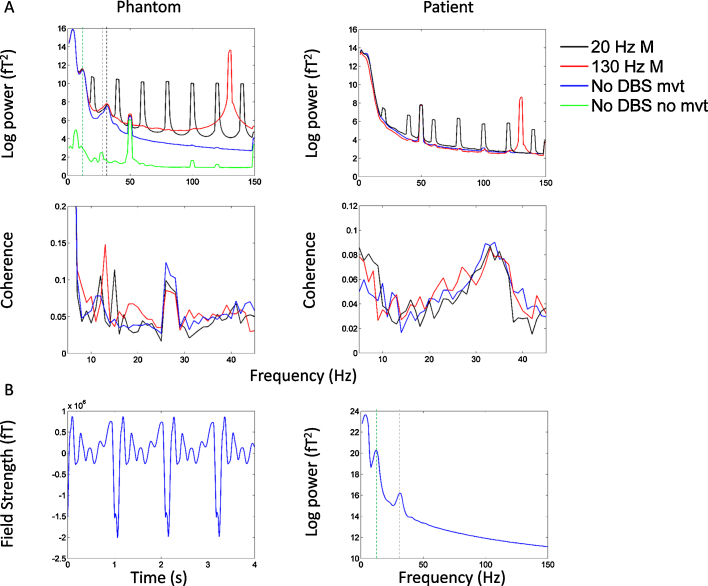
In the top left panel, mean spectra of all MEG channels are shown for the No DBS, 130 Hz monopolar DBS and 20 Hz monopolar DBS conditions in the phantom experiment. For reference, the green line shows mean spectra when the ferromagnetic wires and the heart beat artefact, caused by periodic movement were not present. Channels with jumps have either been excluded, or corrected using interpolation prior to spectral analysis. In the No DBS, 130 Hz monopolar DBS and 20 Hz monopolar DBS conditions there are large spectral peaks at approximately 12 Hz (green dotted line) and at 32 Hz (black dotted line). These spectral artefacts were not observed in corresponding plots from the patient analysis (top right panel), and are related to the damped movement of ferromagnetic wires. Panel 5B shows a single channel with prominent artefacts at 12 Hz and 32 Hz and its corresponding spectra. Note that a spectral peak at 27 Hz, the frequency of the simulated sinusoid, is seen when ferromagnetic wires and heart beat artefacts are not present (dotted grey line) (For interpretation of the references to colour in this figure legend, the reader is referred to the web version of this article.). The small peak at 50 Hz represents power line noise. Monopolar DBS results in a large artefact peak at the stimulation frequency (20 Hz or 130 Hz) and its harmonics. Similar spectra are shown for the bipolar DBS conditions in Supplementary Fig. S1. The middle left and right panels of Fig. 5 display mean coherence between the reference channel and all MEG channels in the phantom and patient experiments for all experimental conditions. In the phantom experiment, where the reference channel is the simulated sinusoidal signal with added noise, there is a clear peak in coherence at the frequency of the simulated sinusoid (27 Hz), which is similar in magnitude for all experimental conditions. In the patient experiment, coherence between the STN LFP and all MEG sensors is maximal at approximately 32 Hz. Furthermore, the magnitude of coherence is similar for all conditions studied.

**Fig. 6:**
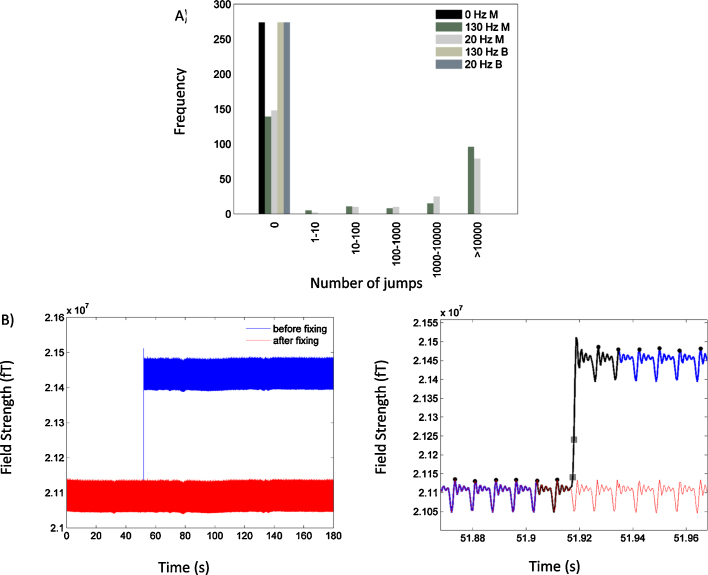
(A) The frequency of the number of jumps in each channel is plotted for the different experimental conditions in the phantom experiment. The stimulation conditions are labelled in the legend by the frequency of stimulation in Hz and either M or B which represent monopolar or bipolar stimulation respectively. Large amplitude jumps were not present during bipolar DBS at 130 Hz or 20 Hz or when DBS was off (see black bar), but appeared during monopolar stimulation at both 20 Hz and 130 Hz. B: The plot on the left hand side shows the signal recorded by a single MEG channel before (blue) and after (red) fixing of a segment containing a single jump, using the interpolation technique described in Section 2. The plot on the right hand side shows a segment of data surrounding the jump. The blue and red lines display the data before and after fixing of the jump. The black line represents the data segment that is interpolated and the grey squares show samples either side of the first jump within the region encompassed by the black line. Interpolation involved averaging data segments between stimulation pulse peaks (shown by the black circles) as described in Section 2. (For interpretation of the references to colour in this figure legend, the reader is referred to the web version of this article.).

We characterised the time and spectral domain components of DBS artefacts. Spectral analysis of individual channel data was performed by pre-multiplying the data with a hanning taper prior to spectral decomposition using the Fast Fourier Transform. Further details on the artefacts may be found in Section 3 and in Fig. 3, Fig. 4, Fig. 5, Fig. 6.

We have previously defined methodology for determining the statistical significance of resting coherence between a bipolar contact pair of the STN DBS electrode and all MEG sensors (Litvak et al., 2011a). This approach allows us to define frequency bands for subsequent source space analysis in individual patients. In short, we computed coherence between the STN 0-2 bipole and all MEG channels between 5 and 45 Hz with 2.5 Hz resolution. Scalp maps of coherence at each frequency were linearly interpolated to produce 2D images. These 2D images were then stacked to produce a single 3D image having 2 spatial dimensions and 1 frequency dimension (Kilner and Friston, 2010). Ten surrogate images were also generated for statistical comparison, using the same approach, but with the order of the STN-LFP trials shuffled. The original and shuffled images were smoothed with a 10 mm × 10 mm × 2.5 Hz Gaussian kernel and subjected to a two sample *t*-test using standard SPM analyses (Litvak et al., 2011b, Kilner et al., 2005). The SPMs were thresholded at *p* < 0.01 (family wise error corrected) to enable the identification of significant regions in frequency and sensor space.

### 2.13 Source space analysis

Sources coherent with the reference channels were localised using DICS beamforming (Gross et al., 2001) as described in our previous publications (Litvak et al., 2010, Litvak et al., 2012, Oswal et al., 2013b, Oswal et al., 2014). Beamforming rests on a linear projection of sensor data using a spatial filter that is computed from the leadfield of a location of interest and either the data covariance or the cross-spectral density matrix (Gross et al., 2001, Van Veen et al., 1997).

For the phantom lead fields were computed using a single sphere model coregistered to MEG sensors based on HPI coil location measurements. For the patient we used a single shell head model (Nolte, 2003, also called corrected-sphere model in more recent literature). The model was generated in SPM based on the patient's pre-operative structural MRI and fiducial-based co-registration was performed as described in Litvak et al. (2010).

The source space was defined as a 5-mm-spaced grid limited to the inside of the sphere for the phantom or to the inside of the skull compartment for the patient. For the phantom experiment, DICS beamforming was used to provide estimates of coherence in the 26–28 Hz band between the simulated sinusoidal signal and each point spaced on a 5 mm spaced grid within the phantom. Since the simulated signal that was recorded by the BrainAmp had an unrealistically large coherence with the same signal recorded by MEG, we added random Gaussian noise with fixed RMS amplitude to the recorded simulated sinusoidal signal in order to reduce the coherence to a more physiologically plausible value. No noise was added to the MEG signal, however.

The goal of this particular analysis was to generate a 3D image showing coherence between the simulated sinusoidal signal recorded with the BrainAmp and the spherical volume encompassed by the phantom. This is the kind of analysis that has been typically applied to patient data in our previous studies. A similar approach was also used for the single subject data we present, with a few important differences as discussed below. Firstly, for the patient experiment, DICS beamforming was used to provide coherence estimates in the physiological frequency range that was identified as being significant in sensor level analysis. Secondly, the reference signal used was the bipolar output of the stimulation-record amplifier, representing the potential difference between contacts 0 and 2 of the inserted left DBS electrode. Thirdly, for source extraction in the patient analyses we defined the beamformer source orientation as the normalised imaginary part of the cross-spectral density vector between the recorded LFP and the *x*, *y* and *z* orientations of the MEG source (Nolte et al., 2004, Litvak et al., 2010). This helps to focus on the cortical signal component coherent with a delay to the subcortical LFP, which might not be the highest amplitude component at each location. In the case of the phantom experiment, however, the simulated dipole signal was the only signal in the system and there was no delay between it and the reference. Therefore, we just used the orientation of maximum power (Gross et al., 2001).

The resulting coherence values were then linearly interpolated to produce 3D volumetric images with 2 mm resolution for visualisation. Importantly, common spatial filters were used to generate images for each of the three different experimental conditions. Common spatial filters were generated from the data covariance matrix computed using all experimental conditions. These filters were then applied separately to the data for each experimental condition in order to allow comparison of experimental conditions. In both the phantom and patient experiments a beamformer regularisation parameter of 0.01% was used. Regularisation corresponds to whitening of the data covariance matrix by adding an identity matrix multiplied with a constant (Brookes et al., 2008). We have previously shown empirically that this degree of regularisation provides optimal artefact suppression in resting patient recordings (Litvak et al., 2010). Once a single coherence image had been generated for each stimulation condition in the phantom experiment, the images were averaged in order to select a single peak location for time series extraction using LCMV beamforming (Van Veen et al., 1997). Importantly, in the patient experiment we were not interested in generating DICS images for each stimulation condition, but instead wanted to determine how resting STN-cortical coupling is influenced by DBS. Accordingly we generated a DICS image of STN-cortical coherence only in the no DBS condition.

Having identified a location of peak coherence, source time series were extracted from this location using LCMV beamforming. Common filters were used for the three different experimental conditions with regularisation parameters as described above for the DICS beamforming stage. Once individual trial time series were extracted for the three different experimental conditions, coherence was computed between the reconstructed source and the reference channel using multitaper spectral estimation with a frequency resolution and taper smoothing frequency of 2 Hz (Mitra and Pesaran, 1999). The power spectrum of the reconstructed source was also computed using the same approach. In the case of patient recordings, the source time series were extracted from the DICS image peak. In order to provide additional immunity from artefacts robust averaging was applied to both the cross-spectral density estimates and the auto-spectra across trials prior to computing coherence. In essence robust averaging is a special case of the robust general linear model (Wager et al., 2005) and involves down weighting the contribution of outlier trials that are contaminated by artefacts. Further details and a validation of robust averaging in a context similar to our present analysis can be found in (Litvak et al., 2012).

### 2.14 Nonparametric statistical testing of coherence/power differences

Finally, for the phantom data we performed statistical comparison of the differences in coherence between the three conditions. We used non-parametric permutation testing, whereby individual trials of the two time series were randomly assigned to one of the three condition labels prior to computing coherence. One thousand such permutations were employed, giving rise to a null distribution of absolute condition-specific coherence differences. In order to control for there being multiple (three) pairwise comparisons (no stimulation vs. 20 Hz, no stimulation vs. 130 Hz and 20 Hz vs. 130 Hz) we constructed the null distribution from the maximal absolute coherence difference between the three conditions at each permutation, following the procedure described by (Ernst, 2004). Following construction of the null we used a significance level of *α* = 0.05, in order to check for significant differences in coherence between the three experimental conditions. For statistical analyses in the phantom experiment we were only interested in testing for significant differences in coherence between conditions at 27 Hz since this was the frequency of the simulated signal. The same approach was used to test for statistically significant differences in power.

## 3 Results

### 3.1 Stimulation related artefacts

Raw data recorded in the absence of DBS in the patient showed artefacts related to the movement of ferromagnetic wires due to arterial pulsations caused by the heartbeat. This type of artefact has been described in detail in (Litvak et al., 2010) and was realistically simulated in our phantom recordings. The upper panel of Fig. 3 shows individual channel power spectra for the three experimental conditions in the phantom recording. Common to all three conditions is the presence of high amplitude low frequency artefacts (<10 Hz), which affect the majority of channels and are caused by the movement of ferromagnetic components. Note that the spectra for the two stimulation conditions contain fewer channels, since channels with large number of jumps have been rejected. In order to establish the temporal characteristics of the low frequency artefacts, we present data from a single channel and a 3 s long recording period with the highest power in frequencies less than 10 Hz. Data are presented in the lower panel of Fig. 3 for the no DBS condition and the two monopolar DBS conditions. The left hand side plots (Panel A) show 3 s of data with the same *y* axis for comparison of the signal amplitudes. The middle plots (Panel B) show only 0.08 s of data and the *y* axes are varied so that the signal can be visualised in all 3 conditions. The plots on the far right are the spectra of the data presented in Panel A. The effects of movements of the ferromagnetic wires which occur at a rate of approximately 1 Hz are clearly visualised in Panel A. Furthermore, the effects of ferromagnetic wire movement appear similar across the three different stimulation conditions.

Data recorded during stimulation in both the patient and the phantom showed two additional kinds of artefacts: (1) DBS pulses followed by ringing (2) jump artefacts. The first type of artefact was observed during both bipolar and monopolar DBS, whereas the second type of artefact were observed only during monopolar DBS. In the following paragraphs we will describe these artefacts in further detail.

Firstly, each DBS pulse produced a large artefact followed by a ringing response in all channels whose amplitude ranged from hundreds to several thousands of femtoTesla (fT). This ringing arises from the response of the antialiasing hardware filter in the CTF machinery to a stimulation pulse. In our recordings, with a sampling frequency of 2400 Hz, the antialiasing filter was an 8th order elliptic low-pass filter with a cut off of 600 Hz. This filter resulted in a ringing decay time constant of 4.8 ms. As a result, most of the period between stimulation pulses (7.7 ms) was affected by ringing during both monopolar and bipolar DBS at 130 Hz (see right hand side panel of Fig. 4). In contrast, the period between stimulation pulses at 20 Hz is longer (50 ms) and therefore less affected by ringing (see left hand side panel of Fig. 4). In comparing the upper and lower panels of Fig. 4, it is worthwhile noting that the magnitude of the artefacts induced by monopolar DBS at both stimulation frequencies was an order of magnitude (approximately 40×) greater than that induced by bipolar DBS. Although the antialiasing filter had an undesirable side effect of introducing severe ringing artefacts, it prevented the emergence of spurious peaks in the power spectrum, generated by aliasing of stimulation frequency harmonics above the Nyquist frequency of 1200 Hz (please see Section 4 for further comment on this).

Fig. 5A shows the power spectrum averaged across all MEG channels for the no DBS and monopolar DBS conditions in both the phantom experiment and in the patient experiment. Note that channels with jumps caused by DBS had either been corrected or excluded from all conditions prior to plotting. For sake of clarity spectra for the bipolar DBS conditions in the phantom experiment are shown in separate figure, supplementary Fig. S1. These spectra show a number of important features. Firstly, in the case of the phantom experiment a peak at 27 Hz representing the simulated sinusoid is not observed due the presence of strong background noise caused by the movement of ferromagnetic wires. Note that very similar spectra are observed in the patient recording. The green line in Fig. 5A shows the mean spectrum for the phantom recording when the ferromagnetic wires are removed and heartbeat artefacts are not simulated. In this case it is possible to see a small peak in the frequency spectrum at 27 Hz (indicated by the grey dotted line). We note that there are large spectral peaks in the phantom recordings at approximately 12 Hz (green dotted line) and at 32 Hz (black dotted line). These spectral artefacts were not observed in the patient recording, and are related to the damped movement of ferromagnetic wires. Panel 5B shows a single channel with prominent artefacts at 12 Hz and 32 Hz and their corresponding spectra.

Crucially in Fig. 5A we display mean coherence between the reference channel and all included MEG sensors. In the case of the phantom experiment, where the reference channel was the simulated sinusoidal signal at 27 Hz, a coherence peak at this frequency is clearly visualised and is similar in magnitude for the three experimental conditions. In the case of the patient experiment, where the reference channel was the STN LFP a peak in coherence at approximately 32 Hz is seen which is once again similar across all experimental conditions.

Common to the patient and phantom recordings is the presence of a peak at 50 Hz representing power line noise. During DBS at 20 Hz, there are high amplitude peaks at 20 Hz and its harmonics (40 Hz, 60 Hz, 80 Hz, 100 Hz, 120 Hz, 140 Hz). Comparing monopolar (black line in Fig. 5) with bipolar (black line in Supplementary Fig. S1) DBS at 20 Hz, it is evident that the amplitudes of the 20 Hz peak and its harmonics are markedly lower with bipolar DBS. A similar pattern is also observed for monopolar (red line in Fig. 5) and bipolar (red line in Supplementary Fig. S1) DBS at 130 Hz. A more detailed analysis of the reasons for differences between monopolar and bipolar DBS is reserved for Section 4.

The second type of artefact we noted was jumps affecting a subset of the channels during monopolar DBS at either 20 Hz or 130 Hz. Jumps comprised abrupt changes in the signal level with a fixed amplitude of ∼3.5e5 fT (there are differences between individual channels depending on their calibration), followed by a ringing artefact as described above. This value corresponds to *ɸ*_0_—one quantum of magnetic flux in the CTF SQUID sensors. This indicates that the jumps resulted from the inability of SQUID electronics to lock to the DBS pulse due to its very fast rise time. Different MEG channels varied in the number of jumps they contained. Fig. 6A is a histogram of the number of channels affected by different numbers of jumps at the different stimulation conditions. Data are shown for the phantom recording, and similar features were present in the patient data. The majority of the 275 channels recorded during monopolar DBS were not contaminated by jumps (139 and 148 channels were jump free in the 130 Hz and 20 Hz monopolar DBS conditions respectively). Some channels had only a small number of jumps, but there were also channels with a very large number of jumps where the recording over the whole session looked like a staircase. Since channels with a large number of jumps were only a minority and the amount of useful signal potentially recoverable from them by any correction method appeared to be small, we excluded these channels from further analysis (see Methods). For the remaining channels we used an interpolation approach to remove the jumps and preserve the rest of the recording. In order to illustrate that this approach suppresses discontinuities well, we selected a single channel with between 100 and 1000 jumps in both DBS conditions (130 Hz monopolar DBS and 20 Hz DBS) and statistically compared the difference in the amplitudes of DBS pulse peaks situated either side of a corrected jump with the difference in amplitude of adjacent DBS pulse peaks that were not intervened by jumps. In both stimulation conditions a two sample *t*-test suggested the absence of statistically significant differences (130 Hz monopolar DBS condition: *t* = 0.16, df = 1058, *p* = 0.16; 20 Hz monopolar DBS condition: *t* = 0.21, df = 1005, *p* = 0.09). This procedure enabled us to recover 30 channels in the phantom experiment and 35 in the patient experiment. Fig. 6B shows single channel data, for a channel with 1 jump at a stimulation frequency of 130 Hz before (blue line) and after (red line) fixing of the jumps using the interpolation-based approach described in Section 2. On the right hand side of Panel B we have zoomed into the region surrounding the third jump. Samples either side of the detected jump are indicated by the grey squares and the region affected by the jump, which is interpolated is shown in black. The original data and the fixed data are shown in blue and red respectively.

Although our experiments revealed that bipolar DBS resulted in fewer artefacts than monopolar DBS (see Section 4), we were unable to perform simultaneous stimulation and recording of the STN during bipolar DBS as the common mode rejection of our stimulation record amplifier required that the two recording contacts symmetrically sensed the stimulation signal. In the following sections we, therefore, restrict our analysis to the effects of monopolar DBS. We first show using the CTF current dipole phantom that it is possible to accurately recover coherence between a reference channel and an MEG signal in source space, using beamforming, despite the presence of monopolar DBS related artefacts.

### 3.2 Phantom results

**Fig. 7:**
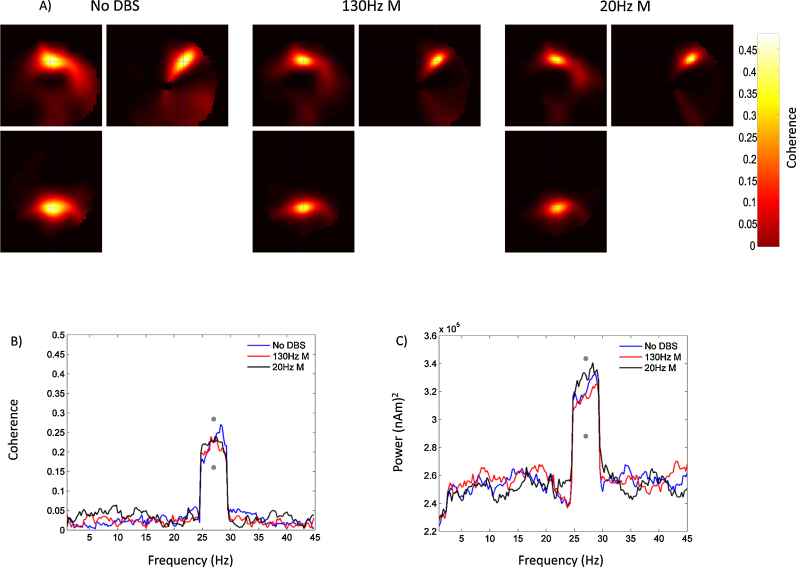
Panel A shows DICS beamformer images of coherence between the simulated LFP and a 5 mm spaced grid within the phantom. These images were interpolated, as described in Section 2, giving final images with 2 mm resolution. Coherence images are shown for the three different experimental conditions: no DBS, monopolar DBS at 130 Hz and monopolar DBS at 20 Hz. In all cases the coherent source was clearly visualised, and the coherence values (see colour bar) were comparable between the three different stimulation conditions. The intersection of the cross-hairs indicates the location of the peak value of coherence, which was identical across experimental conditions. Panel B shows coherence between the simulated LFP and LCMV beamformer extracted source time series from the location of peak coherence indicated by the cross-hairs in Panel A. Coherence is plotted as a function of frequency for the three experimental conditions. The grey dots indicate 95% confidence intervals of the null distribution of condition specific coherence differences at 27 Hz generated by permutation testing. For sake of visualisation, this confidence interval has been added to the absolute value of coherence at 27 Hz for the no DBS condition. All of the observed differences in coherence between stimulation conditions lie within this limit, highlighting the absence of statistically significant differences. Panel C shows a power spectrum of the extracted source time series with the grey dots representing 95% confidence intervals of the null distribution of condition specific power differences at 27 Hz which have been added to the absolute value of power at 27 Hz for the no DBS condition.

Fig. 7A shows DICS beamformer source space images of coherence for the phantom experiment during the no DBS and two monopolar DBS conditions. As discussed in Section 2, coherence was computed in the 26–28 Hz band between the simulated sinusoidal signal with added random noise and each point on a 5 mm grid within the phantom. A clear peak in coherence (shown by the intersection of the cross-hairs), corresponding to the location of the simulated dipole, is observed at each of the different stimulation conditions and the absolute values of coherence are comparable across conditions. Panel B of Fig. 7 shows coherence between the simulated sinusoid with added noise and the timeseries extracted from the peak of the DICS images for the three different experimental conditions. At 27 Hz, the frequency of the stimulated sinusoidal signal, coherence values for the three stimulation conditions are statistically indistinguishable. This is highlighted by the fact that the observed differences in coherence between the three conditions at 27 Hz do not fall within the 5% extremes of the constructed null distribution, indicated by the grey dots in Fig. 7B. These results highlight that it is possible to accurately recover simulated coherence in the presence of high amplitude artefacts caused by monopolar DBS. The accurate recovery of simulated coherence is due to the presence of consistent phase relationships between the simulated sinusoid and its activity represented at the MEG sensors and the lack of such consistent phase relationships between the simulated sinusoid and DBS related artefacts. In Fig. 7C, we also display a power spectrum of the time series extracted from the peak of the DICS image for the three different experimental conditions. At the frequency of the simulated sinusoidal signal (27 Hz) power values are statistically indistinguishable for the three stimulation conditions - highlighted by the fact that the observed differences in power do not fall within the 5% extremes of the constructed null distribution. Furthermore, an important feature of this plot is that low frequency power artefacts observed at sensor level (see Fig. 3, Fig. 5) are quite well suppressed by source space analysis through beamforming.

### 3.3 Single subject results

**Fig. 8:**
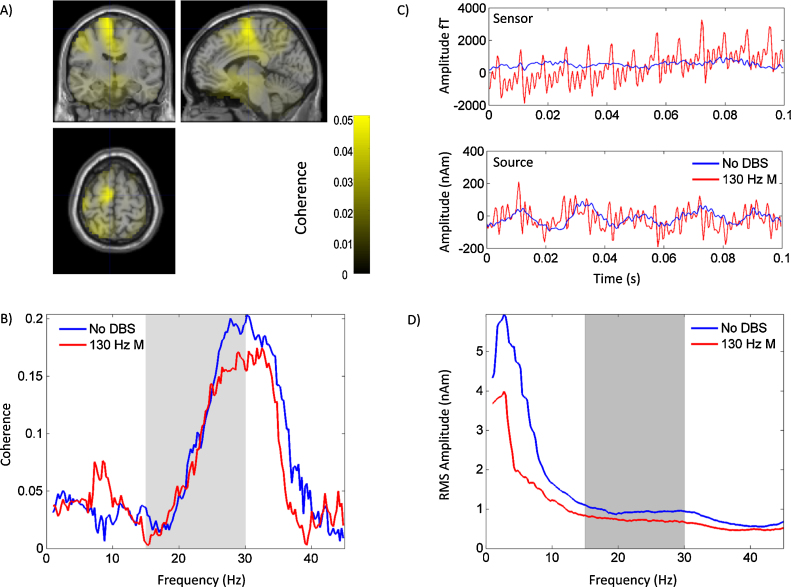
The results of the patient analysis are shown. Panel A shows a DICS beamformer image of coherence in the beta frequency (15–30 Hz) range between the left STN 0-2 bipole and the whole brain when the patient is at rest without DBS. The unthresholded DICS coherence image is superimposed onto a T1 weighted canonical MRI. Coronal, sagittal and axial sections through the image are displayed. The intersection of the cross-hairs represents the location of peak coherence (MNI co-ordinates -6 -10 66 corresponding to the premotor/supplementary motor areas). Source time series are extracted from the peak location and source RMS amplitudes and STN-cortical source coherence are computed for the no DBS and the 130 Hz DBS conditions. The results are shown in Fig. 8D and B respectively. The grey region indicates the beta frequency range which was used to determine the location of the cortical source. Finally panel C shows a 0.1 s long segment of data recorded from a single MEG channel and from the source time series in the no DBS and the 130 Hz monopolar DBS conditions. It is evident that the sensor level artefacts that are observed during DBS are quite well suppressed by beamforming.

The patient had a good clinical response to DBS, with total off medication UPDRS part III motor scores improving from 63 to 30 with the introduction of bilateral STN DBS at 130 Hz. Jump artefacts, ringing and low frequency artefacts observed in the phantom were also observed in the patient experiment and the channel power spectra of the patient recording were broadly similar to those of the phantom recording with the exception of additional artefactual spectral peaks in the phantom recording as described earlier (see Fig. 5). Furthermore, the number of channels affected by jumps was also similar in the patient and phantom recordings. Single subject data are presented in Fig. 8. Fig. 8A shows an unthresholded DICS beamformer image of coherence between the bipolar LFP signal from contacts 0 and 2 of the left STN electrode and cortical regions at rest in the no DBS condition. Coherence was computed over the entire beta band (15–30 Hz) in keeping with our previous studies and also those of others (Litvak et al., 2011a, Hirschmann et al., 2011). The anatomical location of peak resting coherence corresponds to the intersection of the cross hairs at MNI co-ordinates −6 −10 66. This peak was located relatively close to the group peak of beta band coherence identified in our previous studies, at MNI co-ordinates −18 6 58, which corresponds to the location of the supplementary motor/premotor areas (Litvak et al., 2011a). LCMV beamforming was used to extract source time series from the peak of the beta band DICS image, for the no DBS and the 130 Hz monopolar DBS conditions. Power spectra of the source time series are shown in Fig. 8D (the grey bars represent regions within the beta band). Importantly, as per the results of the phantom experiment (Fig. 7) sensor space low frequency artefacts induced by monopolar DBS are not present following beamformer source extraction. In fact low frequency (<15 Hz) activity is markedly higher in the no DBS condition than in the 130 Hz monopolar DBS condition. This is reassuring since there were important differences between the phantom and patient experiments in terms of the potential sources of noise (see Section 4). Fig. 8C displays data from a single MEG sensor (upper plot) and the source extracted time series (lower plot) for the no DBS and the 130 Hz DBS conditions. Sensor level data recorded during 130 Hz DBS are contaminated with artefacts as previously described, which are suppressed by beamforming.

Coherence computed between contacts 0 and 2 of the left STN electrode and source extracted time series for the no DBS and the 130 Hz DBS conditions is shown in Fig. 8B. In the no DBS condition there is a clear peak in coherence towards the upper end of the beta frequency range, and this appears to be suppressed by DBS. This may be in keeping with the suppression of cortical power in the high beta range that is observed in Fig. 8D, but further statistical analysis and comment of DBS effects on cortico-STN coupling are outside the scope of the present paper.

## 4 Discussion

Recording MEG during DBS is probably one of the most challenging applications of MEG in general. The reason for this is that very weak magnetic field changes induced by synchronised neural activity must be detected in the presence of much stronger fluctuations caused by stimulation currents. DBS in the MEG scanner has been done before by several research groups (Ray et al., 2007, Ray et al., 2009, Airaksinen et al., 2011, Airaksinen et al., 2012, Airaksinen et al., 2014, Mohseni et al., 2010, Mohseni et al., 2012, Cao et al., 2015). Ramírez et al. (2011) also used a phantom to study DBS artefacts in MEG. We will now discuss several important issues pertaining to our present study in the context of previous studies combining DBS and MEG recordings.

### 4.1 Implanted vs. externalised patients

In all previous DBS MEG studies, patients were recruited after stimulator implantation and therefore had a stimulator implanted in their chest wall and no externalised wires. The artefacts observed in such patients are different to those we have described. Heart-beat locked artefacts from ferromagnetic wires in the head are likely to be reduced in implanted patients (although we have not done the comparison ourselves). The reason for this is that a major source of this artefact is the percutaneous externalisation wire that moves with arterial pulses on the scalp (Litvak et al., 2010). This percutaneous wire is removed once the stimulator is implanted and the wire that subsequently connects the DBS electrode to the stimulator is neither ferromagnetic nor as mobile, since it is buried in the subcutaneous tissue.

On the other hand the implanted stimulator in internalised patients is ferromagnetic and hence may give rise to breathing and heart beat related movement artefacts even when it is turned off. When the stimulator is on, the currents flowing inside it induce magnetic field changes that affect the MEG sensors for both monopolar and bipolar stimulation. Since the fields generated by the stimulator have spatial patterns clearly distinct from those coming from inside the head, denoising methods making use of spatial topographies such as Signal Space Separation (SSS) (Taulu et al., 2004), temporal Signal Space Separation (TSSS) (Taulu and Simola, 2006) or S^3^P (Ramírez et al., 2011) can remove them effectively. In our study we implemented two different sensor level denoising approaches, S^3^P (Ramírez et al., 2011) and pTSSS (Sekihara and Nagarajan, 2014)—the latter being a variant of TSSS. The details and results of our implementation are described in the Appendix. We found that applying either S3P or pTSSS did not enhance the ability to recover source power or coherence, as compared to beamforming. Both pTSSS and S3P did however suppress low frequency artefacts and stimulation related artefacts in sensor level analyses.

For our study we recruited patients who underwent DBS surgery in two stages. In the first stage only the intracranial electrodes were implanted and the contacts were externalised for recording using a stainless steel percutaneous extension wire. Only later, a few days after our recording session, was the stimulator implanted. Thus, our patients did not have a stimulator in the chest but did have ferromagnetic metal parts on the head. It is possible to use non-ferromagnetic titanium wires, which are MEG compatible and induce fewer artefacts, but these have not yet been clinically approved for use in the UK. In any case using titanium wires would not eliminate stimulation-related artefacts which are the focus of this paper, but only heart beat and movement-related artefacts that we have previously shown to be effectively suppressed by beamforming (Litvak et al., 2010, Litvak et al., 2011a, Litvak et al., 2012, Oswal et al., 2013b).

The external stimulator used as part of our setup (see Fig. 1) was inside the magnetically shielded MEG room but much further away from the MEG helmet than an implanted stimulator would be (about 1.5 m). The reason for not placing the stimulation equipment outside the shielded room was to ensure that the clinician administering DBS could observe the patient closely at all times for safety purposes. Although the stimulator and stimulation record amplifier will contribute to the MEG signal when they are turned on, this contribution appears to be negligible in relation to the stimulation artefacts in our data.

### 4.2 Monopolar vs. bipolar stimulation

Stimulation-induced magnetic fields that are recorded by the MEG system as artefacts originate from currents flowing in the loop including the stimulation equipment, the wires and the patient. Total magnetic flux generated by this loop is proportional to the area bounded by the current paths. In the case of bipolar stimulation the current flows to and from the patient through intertwined wires and the only place where it leaves the wires is the stimulation site where it is carried by charged ions between two contacts located just 0.5 mm apart. Although some fraction of the current takes a longer path through the brain tissues according to the distribution of impedances, this fraction appears to be small and only induces weak magnetic flux. The situation is entirely different for monopolar stimulation. In this case the current flows from the stimulated contact in the brain to the reference located on the chest passing through large parts of the head and the upper body, thereby creating a large loop. This explains the striking differences we observed between the artefacts induced by monopolar and bipolar DBS.

In theory, combining bipolar DBS with MEG and LFP recordings would result in fewer artefacts, but that would require a stimulation-record amplifier with an initial stage that is not saturated by the large potential gradient created by bipolar stimulation and is at the same time sensitive enough to record the LFP signal which is weaker by many orders of magnitude. To the best of our knowledge, a stimulation record amplifier that facilitates bipolar stimulation and recording has not been described. In any case, it is perhaps more clinically relevant to consider the case of monopolar DBS since this is often seen as being more clinically effective in terms of ameliorating Parkinsonian symptoms (Deli et al., 2011).

### 4.3 Analyses of coherence vs. other features

The present study is the first to our knowledge to describe simultaneous MEG and LFP recording during DBS. Previous studies reporting DBS during MEG recordings have focussed on the analyses of changes in power of spontaneous brain oscillations (Airaksinen et al., 2012, Mohseni et al., 2012, Cao et al., 2015), changes in cortico-muscular coherence (Airaksinen et al., 2014, Connolly et al., 2012, Park et al., 2009) or differences in sensory evoked potentials induced by DBS (Ray et al., 2009, Airaksinen et al., 2011). Of these three types of studies, analyses of cortico-muscular coherence are the most similar to our study as they also focus on coherence between MEG and a reference channel. However, cortico-muscular coherence and cortico-subthalamic nucleus coherence are distinct phenomena that can be differentially modulated by movement and dopaminergic medication (Hirschmann et al., 2013a).

Thus the present study is more closely related to the previous studies (Litvak et al., 2010, Litvak et al., 2011a, Litvak et al., 2012, Hirschmann et al., 2011, Hirschmann et al., 2013a, Hirschmann et al., 2013b, Oswal et al., 2013b) examining cortico-subthalamic nucleus coherence and its modulation by different experimental factors. As modulations by movement, dopaminergic drugs, severity of the clinical symptoms and tremor have been previously described, studying the effect of DBS is to our mind the next logical step.

### 4.4 Factors affecting the recovery of coherence in the presence of DBS

We have previously demonstrated (Litvak et al., 2010) that cortical signals coherent with the STN can be recovered in the presence of MEG artefacts which exceed the amplitude of physiological MEG signals by several orders of magnitude providing that the MEG system remains in its linear range (e.g., there is no signal clipping). The CTF MEG system is unique in this respect as it possesses a particularly wide dynamic range of up to approximately 7e8 fT which exceeds by far even the range of magnetic interferences induced by monopolar DBS.

However, the system is still limited in its ability to track rapidly changing signals. This is because the measurement of magnetic flux with a SQUID involves a feedback loop implemented with electronics that forces a current into the SQUID to keep the flux constant. If the applied field varies too quickly, the electronics loses lock to the signal which results in jumps of fixed amplitude (on our system 3.3e5 to 3.8e5 fT depending on the channel). The appearance of jumps is also affected by the intrinsic noise of the SQUID. If the external interference and the intrinsic noise are small, no unlocks will occur, while in an intermediate range the jumps will occur occasionally (as in Fig. 6B).

The problem could potentially be mitigated by changing the DBS pulse shape to exclude very high frequencies (or equivalently reduce the pulse rise time). However, the safety and clinical efficacy of such modified pulses has not been demonstrated. Thus we preferred to keep DBS settings close to those used in routine clinical practice and tackle the jump problem by data post-processing. But if there is further interest in combining MEG with monopolar DBS in the future, optimizing both MEG and DBS hardware settings is definitely a promising direction to reduce artefact contamination.

We have dealt with jumps in three different ways: (1) excluding severely affected channels, (2) correcting by interpolation and (3) using robust methods for computing coherence. The vast majority of jumps were contained within the excluded channels.

The second type of artefact we observed were DBS stimulation pulses followed by ringing, the latter being the result of antialiasing filters. The antialiasing filters had a beneficial effect of prevented aliasing artefacts, which have been observed in other DBS MEG studies (Ramírez et al., 2011), but had a side effect of producing ringing, which contaminated most of the temporal interval between pulses in the 130 Hz DBS condition (see Section 3). Crucially in the frequency domain, the ringing artefacts appeared only at the stimulation frequency and did not contaminate our frequency range of interest.

One approach for shortening the ringing artefacts would be to use a much higher sampling rate and hence a higher antialiasing filter cut off frequency, since aliasing would only be observed for frequencies greater than the Nyquist frequency (half the sampling rate). Using a higher cut off frequency for the antialiasing filter has the beneficial effect of shortening the time constant of the ringing artefacts, but comes at a computational cost of having to deal with large volumes of data acquired at high sampling rates.

Despite the presence of the observed artefacts we were able to recover simulated coherence in source space using beamforming in the phantom experiment. More specifically, source space coherence values were similar when DBS was off and artefacts were absent to when DBS was on. It is worth mentioning that although we attempted to make the phantom experiment as similar as possible to a patient recording, there are still some differences. For instance, we used a pure sinusoid as the ‘cortical’ signal, and did not incorporate other competing sources. These factors resulted in unrealistically high coherence with the reference channels that we had to artificially reduce by adding random noise to the latter.

Analysis of single patient data also revealed comparable coherence spectra when DBS was on and off. We will leave a detailed discussion of the effects of DBS on cortico-STN coupling to be the subject of a future report, but it should be noted that both the STN LFP and the cortico-STN coherence may be confounded by a temporary stun effect due to recent surgery. This may lead to baseline changes but should spare the difference between spectra recorded acutely on and off DBS, unless the stun effect were to totally suppress baseline beta activity.

### 4.5 Summary and conclusions

The present study paves the way to using MEG as a means of studying DBS effects on oscillatory connectivity between subcortical stimulation targets and the rest of the brain. We have demonstrated that physiological components of interest can be recovered from MEG data in the presence of DBS artefacts as well as artefacts from ferromagnetic percutaneous wires. Accordingly, the effects of DBS on LFP-MEG coherence can be studied.

## Figures and Tables

**Fig. 9:**
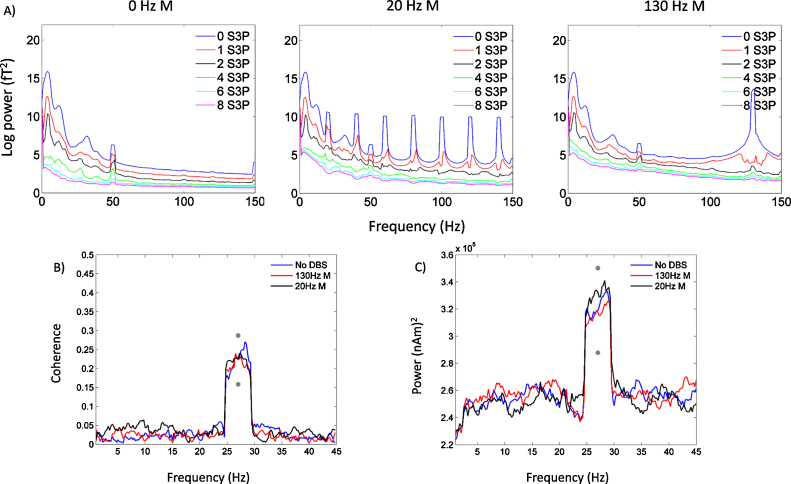
Upper panel (A) shows mean spectra of all MEG channels after removing a fixed number of frequency components using the S3P algorithm. Spectra are shown separately for the no DBS, and two monopolar DBS conditions. Channels with jumps have either been excluded, or corrected using interpolation prior to spectral analysis. Removal of a single spectral component results in good suppression of power at the monopolar DBS frequency (20 Hz or 130 Hz) and its harmonics. Furthermore, this approach can also suppress low frequency artefacts. The lower panel shows the results of performing beamformer analysis following S3P. Note that we decided to adaptively reject components in the two stimulation conditions, such that the power at each frequency could not be less than the power at the corresponding frequency in the no DBS condition. Coherence (B) was computed between the simulated sinusoid with added noise and the beamformer extracted timeseries from the same location as standard beamformer analysis without subspace projection (see Fig. 7). Power spectra (C) were also computed for the source time series. The grey dots represent 95% confidence intervals as per Fig. 7.

**Fig. 10:**
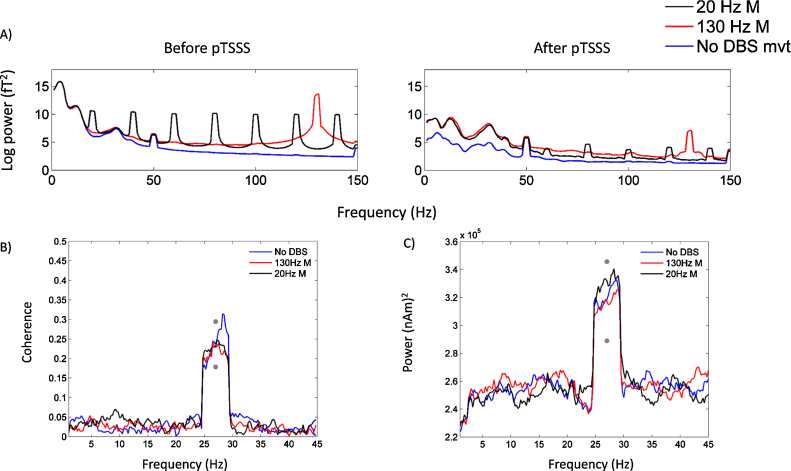
Upper panel (A) shows mean spectra of all MEG channels before and after denoising with the pTSSS algorithm. Channels with jumps have either been excluded, or corrected using interpolation prior to spectral analysis. The pTSSS algorithm results in suppression of both low frequency artefacts and artefacts occurring at the DBS frequency and its harmonics. The lower panel shows the results of performing beamformer analysis following pTSSS. Coherence (B) was computed between the simulated sinusoid with added noise and the beamformer extracted time-series from the same location as standard beamformer analysis without subspace projection (see Fig. 7). Power spectra (C) were also computed for the source time-series. The grey dots represent 95% confidence intervals as per Fig. 7.

## Appendix A **Appendix:** Sensor level denoising approaches

We implemented two different sensor-level denoising approaches to determine whether they could effectively suppress DBS related artefacts in our phantom recordings. The first approach we tested, S3P (Ramírez et al., 2011) involves the generation of time-frequency (TF) specific spatial projectors, which are applied to the noisy TF transformed data. We note that these methods have previously been applied to denoising MEG data recorded during bipolar DBS (Ramírez et al., 2011). The denoised sensor data, may then be obtained through the appropriate inverse transform (e.g. inverse Fourier transform or ifft function in MATLAB) of the projected TF data. Practically, the Fourier transform of the sensor level data yield a complex matrix, *D* ∈ *C*^*nxf*^ where, *n* represents the number of channels and *f* represents each frequency. For each frequency, *f* a cross-spectral density matrix may then be defined as follows, where, *H* denotes the Hermitian transpose:

$$\Sigma_{D}\left(f\right)\;=\;D\left(f\right)D{\left(f\right)}^{H}\;\in \;C^{n\;x\;n}$$

In S3P, eigenvalue decomposition of the complex cross-spectral density matrix is performed yielding frequency specific complex eigenvector and eigenvalue matrices $U\left(f\right)\text{ ∈}\;C^{n\;x\;n}\;\;\text{and}\;S\left(f\right)\text{ ∈}\;R^{n\;x\;n}$ respectively:

$$\Sigma_{D}\left(f\right)=U\left(f\right)S\left(f\right)U{\left(f\right)}^{H}$$

The frequency specific spatial projectors $P\left(f\right)$ are then generated as follows, where *I* represents the identity matrix and *k* denotes the first *k* eigenvectors (or columns of *U*):

$$P\left(f\right)=I-U_{k}\left(f\right)U_{k}{\left(f\right)}^{H}$$

The projected TF data for each frequency is then obtained by applying the frequency specific spatial projector to the original TF transformed data:

$$pD\left(f\right)=P\left(f\right)D\left(f\right)$$

We note that the value of *k* must be specified by the user and we determine the effect of varying *k* on sensor level power estimates. Prior to source space analysis, we also used the no DBS condition as a reference for S3P denoising of the two DBS stimulation conditions. This approach is based on the idea that the power at each frequency is given by the sum of the eigenvalues of the cross-spectral density matrix at that frequency. Accordingly it is possible to select *k* for each frequency of the two DBS conditions, by summing the ordered eigenvalues (*λ* from low to high) of the CSD matrix at each frequency and choosing *k* as the value that minimises the frequency specific difference in power between the no DBS condition and the stimulation condition. Note that *n* represents the number of channels in the following equation:

$$k\left(f\right)=\text{argmin}\left|{\text{pow}}_{\text{No}\;\text{DBS}}\left(f\right)-\overset{n-k}{\underset{i=1}{\sum }}\lambda_{k}\left(f\right)\right|$$

The second denoising approach we tested is similar to the previously described TSSS algorithm, which has been used for denoising MEG data during DBS (Airaksinen et al., 2011, Airaksinen et al., 2014). In standard TSSS a spatial subspace is defined using spherical harmonics in order to separate the data into components arising from within and from outside the head (Taulu and Simola, 2006). A temporal projector is also used in order to project out the unwanted noisy temporal components that are common to two subspaces. The approach we use in this paper, known as pTSSS (Sekihara and Nagarajan, 2014), relies on defining similar subspaces using the singular value decomposition. We had to use this variant of the method because the spherical harmonics based approach is not implemented for the CTF MEG system. Leadfields are first computed for all sources originating inside the head (typically on 5 mm a sampled grid) giving rise to an *n* × *g* matrix, *L* where *g* represents each sampled grid point. A symmetric gram matrix, *G* is yielded by multiplying *L* by its transpose. *G* can then be expressed with its eigenvector, *Ug* and eigenvalue, *Us* matrices as follows:

$$G=UgSgUg^{H}$$

The data *Y* are then projected onto a subspace spanned by the *m* leading eigenvectors of *G* and also to a subspace that is orthogonal to this yielding estimated spatial components that originate from within and from outside the head:

$$Y_{\text{in}}=\left(Ug_{m}Ug_{m}^{T}\right)Y$$

$$Y_{\text{out}}=\left(I-\left(Ug_{m}Ug_{m}^{T}\right)\right)Y=\left(Ug_{n-m}Ug_{n-m}^{T}\right)Y$$

In the analysis presented in this paper we use *m* = 20. The next stage of interference suppression is to remove the common temporal subspace of *Y*_out_ and *Y*_in_. Singular value decomposition (SVD) of the *n* *x* *t* matrices *Y*_out_ and *Y*_in_ (where, *t* represents the number of time points) yields matrices of left singular vectors, singular values, and right singular vectors as follows:

$$Y_{\text{in}}=Uy_{\text{in}}Sy_{\text{in}}Vy_{\text{in}}^{T}$$

$$Y_{\text{out}}=Uy_{\text{out}}Sy_{\text{out}}Vy_{\text{out}}^{T}$$

The first *b* columns of the right singular vectors are used to construct a matrix *Q* whose SVD is used to define a common temporal subspace.

Q=VyinbVTyoutb=UqSqVq

The first *b* right singular vectors $Vy_{{\text{in}}_{b}}$ are projected onto the common temporal subspace, *Uq*.

$$A=Vy_{{\text{in}}_{b}}\left(Uq\right)$$

The *o* leading columns of *A* are used to construct a temporal projector, pT for the data, such that the pTSSS denoised sensor level data are given by, *Y*_clean_ and *I* represents the identity matrix.

$$Y_{\text{clean}}=Y_{\text{in}}\left(I-A_{o}A_{o}^{T}\right)$$

In this paper we used *b* = 20 and *o* = 5.

In the case of S3P we found that both low frequency spectral artefacts related to the movement of ferromagnetic wires and DBS pulse harmonic artefacts could be well suppressed by rejecting a fixed number of frequency components. Mean spectra averaged across channels are shown for the no DBS and the two monopolar DBS conditions before and after S3P for different numbers of rejected components (see Fig. 9). An important problem, however, which this figure highlights is that by rejecting a fixed number of components at every frequency, it is possible to suppress power within a band of interest, in this case at 27 Hz. In order to avoid this problem in further source space analysis, we decided to adaptively reject components in the two stimulation conditions, such that the power at each frequency could not be less than the power at the corresponding frequency in the no DBS condition. The results of beamformer analysis following S3P are shown in Fig. 9B and C. Coherence was computed between the simulated sinusoid with added noise and the beamformer extracted time series from the same location as standard beamformer analysis without subspace projection (see Fig. 7). Power spectra were also computed for the source time-series. Reassuringly, the power and coherence spectra are remarkably similar with (Fig. 9) and without S3P (Fig. 7).

The results of pTSSS pre-processing are shown in Fig. 10. The upper panel of Fig. 10 shows mean spectra averaged across all channels for the three experimental conditions before and after applying the pTSSS algorithm. It is evident that pTSSS suppresses, but does not entirely remove both low frequency artefacts and the DBS pulse artefacts. Importantly, however, beamformer analysis following pTSSS as described above reveals power and coherence spectra that are remarkably similar to those following beamformer analysis alone. These results demonstrate that beamforming effectively suppresses the types of DBS related artefacts that we have observed in sensor space. We have shown that both S3P and pTSSS are useful for denoising sensor level data but they do not offer any additional advantage when performing source space analysis with beamforming.
