# Supplementary material for: Analysis of simultaneous MEG and intracranial LFP recordings during Deep Brain Stimulation: a protocol and experimental validation
Source: J Neurosci Methods. 2016 Mar 1;261:29–46. doi: 10.1016/j.jneumeth.2015.11.029 (PMC4758829; doi:10.1016/j.jneumeth.2015.11.029)
Supplement: Supplementary file 1 [file mmc1.pdf]

Intended Use: BrainAmp amplifiers

BRAIN PRODUCTS
Solutions for neurophysiological research

1. Product definition

BrainAmp products are active medical devices (class IIa) in accordance with Medical Devices Directive MDD 93/42/EEC, Annex IX and include the following versions:

a) BrainAmp Standard
b) BrainAmp DC
c) BrainAmp MR
d) BrainAmp MR plus
e) BrainAmp EXG
f) BrainAmp EXG MR

The intended use of the corresponding medical device also applies to the accessory devices as listed in the relevant Operating Instructions.

2. a) GMDN collective terms (international, Global Medical Device Nomenclature)

I. **CT497** Amplifiers and associated devices
II. **CT643** Power supplies and associated devices
III. **CT384** Sensors and associated devices

b) UMDNS code (Universal Medical Device Nomenclature System Germany, re. I.):
11-467 (GMDN 11467) electroencephalograph.

3. Function description

All the medical devices listed under (1) detect voltage fluctuations by means of active or passive sensors (electrodes, probes). The signals acquired are amplified and then transferred to computer in digitized form for display and storage.

4. Intended use

The devices listed under (1) are intended for medical and non-medical neurophysiological research and are intended for the investigation of physiological processes for human beings and animals. They are not to be used for diagnostic or therapeutic purposes, for the treatment of disease or the conduct of life-preserving, life-monitoring measures.

The devices must be used by appropriate specialist staff or personnel suitable for the utilization of the devices in the application environment as defined by the manufacturer, and in accordance with the Safety and Operating Instructions set out in the relevant manual.

5. Special areas of application

a) MRI environment
Special safety regulations and provisions apply to use in MR scanners. These are defined in the corresponding Operating Instructions. The devices intended for this kind of use are labeled accordingly (MR in green).

MR

b) Invasive EEG
Due to their construction according to IEC 60601-1, BrainAmp amplifiers are type BF applied parts. Our devices' leakage currents permit the use in combination with special electrodes in the central nervous system, being authorized for this purpose by the corresponding manufacturers -
provided that:
- the power supply to the BrainAmp amplifiers shall be ACCU/battery supply only;
- no other devices are electrically connected to the test subject at the same time;
The necessary approval required for the applied electrodes (strips, mats) as a class III /MDD device, must be communicated on request. Brain Products neither specifies nor markets any invasive electrodes.

6. **Authorized associated devices:**

   The accessory devices authorized for each medical device are listed in the corresponding Operating Instructions.

7. **Excluded applications**:
   in accordance with EU Directive 93/42/ EEC (MDD), IEC 60601-1, 3rd edition

   a) The products listed under (1) shall not be used in rooms where explosive gases are present.
   b) The use of a defibrillator while our devices are in operation/connected to the subject is prohibited.
   c) They shall not be used for diagnostic, therapeutic purposes, treatment of disease or the conduct of life-preserving measures.
   d) The use of our devices by laymen, untrained or unsuitable staff as well as use in non-professional environments is prohibited.
   e) Accessory devices which are not authorized for use within the patient environment, are listed in the relevant Operating Instructions (e.g. BrainAmp as of version 014).

8. **Service life**
   The life time of our hardware products is defined as 5 years as of the date of manufacture.

9. **Cleaning**
   Information on cleaning is described in the relevant Operating Instructions. Aggressive or corrosive cleaning agents must not be used.

10. **Maintenance**
    No frequency is specified for safety-related measurements, and such measurements themselves are not mandatory. The users are informed that they shall return the devices if any defect/deficiency is detected.
    Brain Products is solely responsible for repeat testing in accordance with VDE 0751-1/ DIN EN 62353 or for performing the corresponding repairs.

    On request, Brain Products will provide Test Protocols to confirm the continued safety of our devices (IEC 60601-1 based). We recommend to submit the devices for manufacturer inspection every 36 months.
    Users are free to perform an external inspection of the devices and their accessories.

11. **Application environment**

    The following environmental conditions apply for the use of the devices listed under (1):

    Operating conditions: temperature: +10 – 40 °C, relative humidity 25 – 95%, non-condensing.
    Storage: 0 - 60°C, non-condensing
    Air pressure: 700 hPa – 1050 hPa
    Heat, water, moisture, conducting contaminants, extreme levels of radiation may damage the amplifiers and associated devices.

    The basic requirements set out in Directive MDD 93/42/EEC und standard IEC 60601-1 (3rd edition) must be respected, in particular with regard to the patient environment, as well as other applicable national regulations.

Gilching, April 30, 2010

Alexander Svojanovsky
General Manager
