# Supplementary figures and images for: Analysis of simultaneous MEG and intracranial LFP recordings during Deep Brain Stimulation: a protocol and experimental validation
Source: J Neurosci Methods. 2016 Mar 1;261:29–46. doi: 10.1016/j.jneumeth.2015.11.029 (PMC4758829; doi:10.1016/j.jneumeth.2015.11.029)

## Slide 1
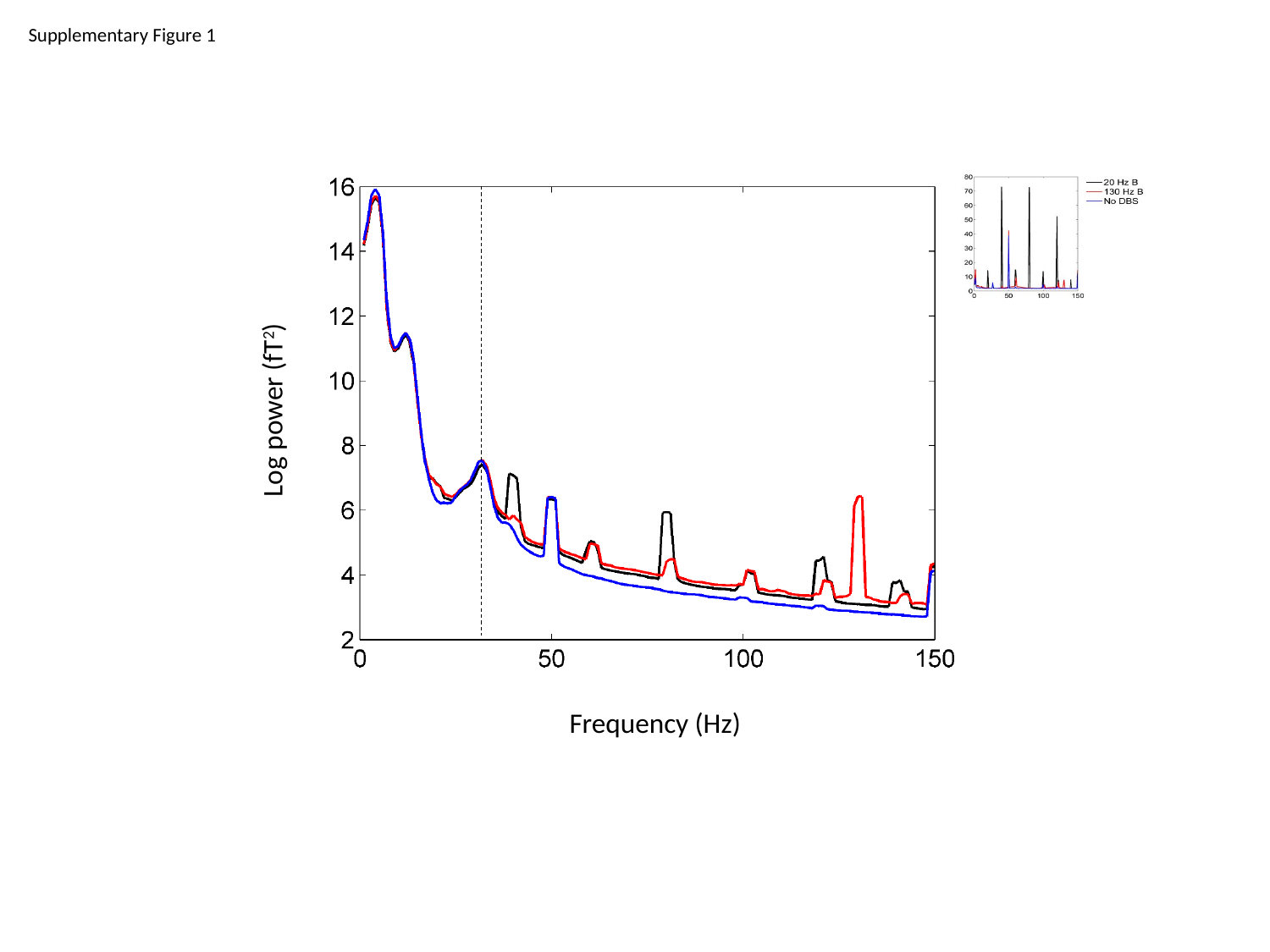

Supplementary Figure 1
Log power (fT2)
Frequency (Hz)

Supplement: Supplementary file 2 [file mmc2.pptx]
